# Workload, Workaholism, and Job Performance: Uncovering Their Complex Relationship

**DOI:** 10.3390/ijerph17186536

**Published:** 2020-09-08

**Authors:** Paola Spagnoli, Nicholas J. Haynes, Liliya Scafuri Kovalchuk, Malissa A. Clark, Carmela Buono, Cristian Balducci

**Affiliations:** 1 Department of Psychology, University of Campania “Luigi Vanvitelli”, 81100 Caserta, Italy; paola.spagnoli@unicampania.it (P.S.); liliya.scafurikovalchuk@unicampania.it (L.S.K.); carmela.buono@unicampania.it (C.B.); 2 Department of Psychology, University of Georgia, Athens, GA 30602, USA; clarkm@uga.edu; 3 Department of Psychology, University of Bologna, 40126 Bologna, Italy; cristian.balducci3@unibo.it

**Keywords:** workaholism, workload, perfectionism, work engagement, job performance

## Abstract

The current study aimed to test how workload, via workaholism, impacts job performance along with the complex interplay of perfectionistic concerns and work engagement in this mediated relationship. A two-wave, first and second stage dual-moderated mediation model was tested in an SEM framework. Results based on a sample of 208 workers revealed a complex and nuanced relationship among the studied constructs, such that the simple mediation model was not significant, but the indirect effect was negative, nonsignificant, or positive conditional on both moderators. The results offer interesting theoretical and practical implications for future studies to be conducted in this area of research. In particular, lower levels of perfectionistic concerns were associated with a positive relationship between workload and workaholism, and lower levels of work engagement were related to a negative link between workaholism and job performance. Findings suggest work engagement should be monitored and promoted by managers, especially when workload, and consequently, the possible risk of workaholism, cannot be avoided.

## 1. Introduction

The issue of what predicts high job performance is one of the most studied in industrial–organizational psychology and human resource management [1]. Changes occurring in work settings and work conditions have stimulated interesting new research avenues that could provide insights for the implementation of organizational interventions aimed at the promotion and maintenance of healthy and efficient workplaces. Specifically, in recent years, several changing working conditions (e.g., strenuous competition, enhanced change and instability, requests for flexibility and continuous learning), combined with the pervasive use of information and communication technology, have placed increased work demands on employees at the expense of other life domains, such as private life and health [2]. A high workload is a common work demand that refers to having too much to do in too little time [3], and it has been shown to have both negative and positive effects on performance [4]. On the one hand, workload could be regarded as a threatening stressor with an adverse effect on performance because it imposes demands on the individual who may not have enough resources (e.g., time) to overcome them. On the other hand, high workload may also occur when high performers take on more tasks and responsibilities and therefore are motivated to perform them well. In this situation, workload can be perceived as a challenge stressor that is positively, rather than negatively, associated with performance [5]. Due to its ambivalent influence on job performance, workload represents a strategic work condition to be “handled with care” by managers.

Beyond workload, research has emphasized that recent changes occurring in work settings, such as overworking culture, might also enhance the proliferation of workaholic employees, that is, “persons whose need for work has become so excessive that it creates noticeable disturbance or interference with his bodily health, personal happiness, and interpersonal relations, and with his smooth social functioning” [6] (p. 4). Since workaholism was first defined by Oates [6] as a “compulsion or uncontrollable need to work incessantly” (p. 11), research in this field has received attention from several scholars. From a clinical perspective, workaholism is considered a true behavioral addiction and, in line with this notion, the term work addiction has been frequently used to identify the phenomenon [7]. Following Andreassen et al. [8] however, in the present study, we will consider workaholism as a synonym of work addiction. Additionally, a consensus has been reached that workaholism is a genuine and persistent problem whose central feature is compulsive overworking [7]. The problem has a prevalence of up to 10% across industrialized countries and is related to impaired psychosocial functioning of clinical relevance [9]. Importantly, it was also acknowledged that although workaholism is closely related to obsessive–compulsive personality, the factors contributing to its development go far beyond personality alone [9]. In line with this, organizational studies have found that although workaholism has been linked to a few positive outcomes, such as career prospects [10,11], it is also related with a variety of negative outcomes, such as an increased risk for metabolic syndrome [12], elevated systolic blood pressure [13], sleeping difficulties [14], work–family conflict [15], and lower relationship satisfaction [16]. In fact, research on workaholism has been particularly focused on the detrimental outcomes for the employees’ health and quality of life in general, and less attention has been given to the relationship between workaholism and job performance.

There does not appear to be a general consensus on the relationship between workaholism and job performance. Some authors have emphasized that workaholics are extremely productive “hyper-performers” [17,18,19,20] and in this case, managers would have an interest in promoting excessive work among employees and establish a reward system that promotes the workaholic behavior. Other scholars have pointed out that workaholics could be poor performers who sacrifice work quality in an attempt to achieve a high quantity of work [21,22]. Accordingly, recent contributions have emphasized that to maintain a high-quality standard of employees’ performance, an adequate recovery period is needed [23,24]. These studies are based on the effort–recovery model [25], which posits that high effort expenditure in response to high workload, especially if coupled with impaired recovery—which may be common occurrences among workaholics—drains individual energy resources and can develop into negative load effects. In the long run, such load effects manifest as losses of function and health impairment, with negative consequences for job performance. Thus, in other words, working too many hours with a compulsive attitude is not only unhealthy, but it could also be ineffective for job performance.

Drawing from the effort–recovery model, in the current study, we aimed to test a comprehensive model including workload, workaholism, and job performance. Both managers and employees can benefit from understanding the effect of workload and workaholism on job performance. Managers should have a clear idea of what interventions to implement for enhancing, instead of compromising, organizational job performance, and employees should be aware of what could hinder their efforts to have a successful career. In order to shed light on these issues, we adopted an interactionist perspective where both job characteristics, such as workload, and individual characteristics, such as perfectionism, were examined as antecedents in the model. Moreover, the effect of work engagement, considered as a possible buffer of the detrimental effect of workaholism, are included in the model. Theoretical and empirical support to our hypotheses is reported in the following paragraphs.

### 1.1. The Relationship between Workload, Workaholism and Performance

Although early workaholism research conceptualized the construct primarily as a stable individual difference characteristic [22], there is accumulating evidence that the organizational context can also shape future workaholism. Ng et al.’s [20] theoretical model of workaholism proposed workaholics’ behavior might be the result of a system of organizational reinforcements. Specifically, if working hard is seen positively within an organization and becomes an important factor in obtaining salary, career advancements, and rewards, employees may be motivated to work harder. Moreover, workaholism could be a dysfunctional coping strategy developed in response to chronically high job demands [26]. That is, an individual constantly facing high job demands may cope by putting more energy and effort into work-related activities and spending progressively more time on them. As a result, work increases in salience and centrality for the individual. Accordingly, Balducci and colleagues’ [13] findings support the view that workaholism may be influenced by certain working conditions, namely, working constantly under time pressure and having many tasks to accomplish (i.e., workload). Additionally, Kanai and Wakabayashi [27] showed that high workload was related to increased levels of workaholism in both white- and blue-collar Japanese employees. Finally, Huyghebaert and colleagues [28] tested a cross-lagged model on a sample of French managers, examining the role of workload in the onset of workaholism by considering the two core dimensions of the workaholism measure, that is, working excessively and working compulsively. Their findings showed that workload did have an influence in predicting working excessively, although it did not significantly predict working compulsively.

In the current study, we adopted the view that organizational conditions, such as having a high workload, can be related to workaholism’s onset. We believe this perspective would be more helpful and interesting for the stemming human resource management implications. In fact, workload could be a strategic work characteristic that could be managed by practitioners, whereas personality traits, such as workaholism, cannot. As Balducci and colleagues [21] suggested, organizational interventions cannot change workaholic tendencies; however, they can create the conditions for them to remain silent.

Turning next to the relationship between workaholism and job performance, the issue of a possible negative influence of workaholism on job performance still seems to be controversial. However, such negative influence becomes understandable if one considers reduced performance as a manifestation of the impaired social functioning which is attributable to workaholism [7]. Some recent contributions support that workaholism has a negative influence on job performance, while other studies report a null relationship. For instance, Falco and colleagues [29] found a positive relationship between workaholism and psychophysics strain, which in turn was negatively associated with job performance. Gorgievski, Moriano and Bakker [30] reported that workaholism was related to negative affect, which in turn related negatively to entrepreneurs’ performance. More recently, contrary to their expectations, Balducci and colleagues [21] found a null effect of workaholism on job performance measured one year later. This latest study would support Clark and colleagues’ [11] meta-analysis findings, reporting a null relationship. Perhaps then, since studies suggest that organizations rather appreciate workaholic behaviors and work addiction is related to higher managerial positions, performance evaluations may be biased if productivity is based on supervisors’ performance ratings using subjective Likert-type scales.

At a broader level, one reason for the equivocal results may be the differences in how workaholism is conceptualized across studies. In fact, the earlier conceptualization of workaholism also included work enjoyment [22], whereas, currently, a general consensus exists among researchers in conceiving workaholism as a negative phenomenon [11]. According to Schaufeli, Taris and Bakker [31], workaholics seem to work hard rather than smart. They create difficulties for themselves and their colleagues, are rigid, inflexible, and perfectionist, as well as not inclined to delegate. This, in the long run, can create conflict and friction in the workplace, which results in low social support and, subsequently, low job performance. Thus, a negative relationship between workaholism and job performance would be expected. Taken together, more research on the relationship between workaholism and job performance is still needed to shed light on this issue. In the current study, we aimed to contribute to this research stream by testing the relationship between workaholism and job performance in a more comprehensive mediation model where workaholism mediates the relationship between workload and job performance measured one month later. Thus, our first hypothesis is as follows:

**Hypothesis** **1** **(H1).** *Workaholism mediates the relationship between workload and job performance, such that workload positively relates to workaholism, which, in turn, negatively relates to job performance.*

### 1.2. The Interplay between Workload and Perfectionism on Workaholism

Several studies have shown that individual (i.e., personality traits) and situational (i.e., work-related) factors are both involved in the onset of workaholism [11,13,32,33,34,35,36,37]. One of the most important individual factors in predicting workaholism seems to be perfectionism [11,22,38,39,40]. This is understandable since workaholism is closely related to obsessive–compulsive personality disorder and a perfectionistic tendency is an important ingredient in such disorder [9]. Perfectionism can be defined as a tendency of striving towards high personal standards and concerns regarding the extent to which these standards are realized [41,42]. Since perfectionism is characterized by striving for flawlessness and setting exceedingly high, usually unrealistic standards of performance, together with an overly critical evaluation of one’s own behavior [43,44,45], individuals who are high in perfectionism may spend an excessive amount of time on work, because they tend to be inflexible and rigid about their desired level of performance, and endorse several work-related irrational beliefs as all-or-nothing judgment of their performance [46,47], which in turn may lead to workaholism [48]. In fact, workaholics work beyond what is reasonably expected from them [49], because of inner compulsion towards working hard [6,50] that could well be the result of a high level of perfectionism [22,49,51]. Additionally, as shown by Aldahadha [52], both types of perfectionism (positive perfectionism and negative perfectionism) were strongly associated with work addiction.

As we have proposed in the previous section, we expect that workload will be positively related to workaholism. However, since a more comprehensive and interactionist view of the onset of the workaholism would be desirable and recommended (see also [9]), we considered perfectionism as one of the most critical personality dispositions for becoming workaholic, and thus, we believe that the combined effect of workload and perfectionism could be the most typical condition for the onset of workaholism. Following this rationale, a previous study conducted by Girardi, Falco, De Carlo, Dal Corso and Benevene [53] reported the moderating role of workload in the relationship between two aspects of perfectionism and workaholism, that is, socially-prescribed perfectionism and self-oriented perfectionism. Moreover, the results provided by Falco and colleagues [54] in their most recent study showed that workload moderated the longitudinal association between self-oriented perfectionism and workaholism, while the interaction between workload and socially-prescribed perfectionism was not significant. Perfectionism is generally considered to be a multidimensional construct [47,55]. Although various dimensions have been proposed and studied, there is a general consensus that two major factors underlie different dimensions of perfectionism: perfectionistic concerns and perfectionistic strivings [55]. Perfectionistic concerns include a family of traits involving generally problematic tendencies or characteristics, such as excessive concerns about making mistakes and chronic disappointment in the self for not living up to high-performance expectations. In contrast, perfectionistic strivings include a constellation of traits involving high-performance expectations or high personal standards. In their meta-analysis, Clark and colleagues [11] found a large positive correlation between perfectionism and workaholism. However, perfectionism was only examined at the overall level, which does not address the issue of possible differential associations for the two perfectionism’s components. In fact, a recent meta-analysis conducted by Harari, Swider, Steed and Breidenthal [56] suggests the relationship between perfectionistic concerns and workaholism is much stronger than the relationship between perfectionistic strivings and workaholism. Based on these findings and the rationale that workaholism is often viewed as a compulsion driven by feelings such as being “distressed or guilty” about not working [22] (p. 2), we include just the perfectionistic concerns dimension of perfectionism as a moderator of the relationship between workload and workaholism. Our hypothesis is as follows:

**Hypothesis** **2** **(H2).** *The positive relationship between workload and workaholism is moderated by perfectionistic concerns, such that the relationship is stronger for individuals higher in perfectionistic concerns than for individuals lower in perfectionistic concerns.*

### 1.3. The Interplay between Work Engagement and Workaholism on Performance

According to the heavy work investment perspective [57], work engagement and workaholism are two work-related states that constitute two faces of the same coin, characterized by two elements: long hours of work and heavy effort. In particular, workaholism is based on an addiction to work (an internal, uncontrollable, and stable predictor), while work engagement is an expression of a passion to work (an internal, controllable, and stable predictor). In brief, employees characterized by a high level of work engagement or workaholism both work intensively for many hours; however, the devoted worker (i.e., high engagement) does so with passionate involvement. Research has shown that work engagement is linked to good job performance. For example, Bakker [58] showed that engaged employees performed better than disengaged employees. Moreover, Bailey, Madden, Alfes and Fletcher [59] claimed that the positive emotions experienced by engaged employees are related to a broader scope of attention and to an ability to build up one’s resources. These resources may include physical resources (e.g., health), social resources (e.g., social support networks), intellectual resources (e.g., knowledge, executive control), or psychological resources (e.g., self-efficacy, optimism). These personal resources can be used to cope with job demands and to perform well [60,61]. Furthermore, work engagement has been found to predict good health [62], and good mental and physical health has been found to predict employee performance [63] as well as long-term financial business performance for the self-employed [64,65]. According to the job demands–resource model, work engagement plays a key role in the motivational process, which links job resources with positive organizational outcomes via work engagement [66].

Workaholism and work engagement differ in their relationship with various indicators of well-being and job performance [67]. For instance, in their cross-sectional and short-term longitudinal (i.e., 7 months follow-up) studies, Shimazu and colleagues [67] showed that workaholism is associated with (future) unwell-being (i.e., high ill-health and low life satisfaction) and poor job performance, whereas work engagement with (future) well-being (i.e., low ill-health and high life satisfaction) and superior job performance. The recent contribution by Balducci and colleagues [21] included both work engagement and workaholism as separate predictors of job performance, and they found evidence of a significant relationship between work engagement and job performance, whereas a null relationship was found between workaholism and job performance. However, according to Loscalzo and Giannini [68], in some employees, workaholism and work engagement could both be present. They proposed a conceptualization of the interplay between workaholism and work engagement, suggesting that engaged workaholics might be protected from the negative outcomes of workaholism comparing to the disengaged workaholics. In corroboration, empirical support was found that work engagement can buffer against some of workaholism’s detrimental self-reported outcomes, such as work-family conflict, negative affect and emotional exhaustion [14,69], as well as on more objective measures, such as serum levels of the proinflammatory cytokine interleukin-17 (IL-17), a possible biomarker of stress [70]. The specific possible buffering effect of work engagement on workaholism’s poor job performance was addressed by Gillet, Caesens, Morin and Stinglhamber [71] in their person-centered approach. They found that job performance was worse for the disengaged workaholics than for the engaged workaholics. However, their study involved a sample of just teachers and nurses and thus it is unclear how this generalizes to other professions. Thus, drawing from the above theoretical and empirical evidence, in the context of our integrated model, we propose that work engagement could buffer the negative effect of workaholism on job performance measured one month later. We formally hypothesized that:

**Hypothesis** **3** **(H3).** *The negative relationship between workaholism and job performance is buffered by work engagement, such that the relationship is weaker for individuals higher in work engagement than for individuals lower in work engagement.*

## 2. Materials and Methods

### 2.1. Recruitment

Participants were recruited through snowball sampling to participate in an online survey. To help establish temporal precedence and reduce common method variance, a total of 292 participants completed two surveys, separated by 4 weeks. To be included in the study and analyses, participants had to be working at least 30 h per week. After eliminating all the subjects who did not answer the working hours variable and who worked less than 30 h per week, the final sample consisted of 208 participants. On average, participants were 43.9 years old (SD = 11.5, range = 21–65). They had worked for their organization for an average of 15.5 years (SD = 11.2, range = 1–40) and worked about 38 h per week (M = 42.46, SD = 10.7, range = 0–84). They were employees in management positions (5.3%), freelancers (19.4%), teachers (11.2%), healthcare workers (14%), police (5.8%), clerks (42.7%) and other (1.6%). They were evenly split between the private (49.5%) and public sectors (50.5%). Finally, 45.2% were female. 

### 2.2. Questionnaire Administration

Bachelor’s students attending a Work Psychology course in a university in Centre-South of Italy contacted a limited number of available workers to be involved in the study and to forward them the link for filling the online questionnaire. All antecedents, mediators, moderators, and control variables were measured at the first time point while the outcome (i.e., job performance) was measured at the second time point.

### 2.3. Ethics

The procedure was in accordance with the standards of the national law of data treatment, which is strictly followed by the University of Campania “Luigi Vanvitelli” and University of Bologna Alma Mater Studiorum (Italy). Since there was no medical treatment or other procedures that could cause psychological or social discomfort to participants, who were all adult healthy subjects anonymously involved, additional ethical approval was not required. The research was conducted in line with the Helsinki Declaration (World Medical Association, 2001), as well as the data protection regulation of Italy (Legislative Decree No. 196/2003). Participation in the study was voluntary and not rewarded; data collection and analysis were anonymous. A cover letter attached to the questionnaire provided information about the study aims, guarantees about anonymity, voluntary participation and data treatment, and instructions for filling out the questionnaire. When agreeing to fill out the questionnaire, all study participants provided their informed consent by validating the following statement: The participant agrees to the processing of personal and sensitive data collected in the context of this research. The processing of the data collected in the context of the research, their communication to third parties and/or publication for scientific purposes are allowed, but can only take place after the data themselves have been made anonymous, by and under the direct responsibility of the research’s leader.

### 2.4. Measures

#### 2.4.1. Workload

Workload was measured using 3 of the original 5 items in the job demand subscale (e.g., “I have to work very fast”) of the Job Content Questionnaire (JCQ) [72]. We used the Italian version of the JCQ [73], which showed adequate validity and reliability [74] and chose the items with the highest factor loadings according to our use of the scale in other previous studies. Items were asked to be rated on a 5-point scale from strongly disagree to strongly agree. Cronbach’s alpha for the scale was 0.78.

#### 2.4.2. Workaholism

The 7-item Bergen Work Addiction Scale (BWAS) [75] was used to measure workaholism. The BWAS was developed to measure the seven core elements of addiction (i.e., salience, mood modification, tolerance, withdrawal, conflict, relapse, and problems) using one item per element. Example items include “worked so much that it has negatively influenced your health” and “spent much more time working than initially intended” on a 5-point scale from “never” to “always.” Cronbach’s alpha for the scale was 0.78.

#### 2.4.3. Perfectionistic Concerns

Perfectionistic concerns were measured using the discrepancy subscale of the Short Almost-Perfect Scale [76]. This subscale contains four items rated on a 5-point scale from strongly disagree to strongly agree. An example item is: “doing my best never seems to be enough.” Cronbach’s alpha was 0.84.

#### 2.4.4. Work Engagement

The Italian version of the Utrecht Work Engagement Scale (UWES-9) [77] was used to measure work engagement. This 9-item scale contains three subscales (vigor, dedication, absorption) measured with three items each. Example items include: “when I get up in the morning, I feel like going to work” (vigor); “I am enthusiastic about my job” (dedication); and “I am immersed in my job” (absorption). Participants rated items on a 5-point scale from never to always. Cronbach’s alpha was 0.90.

#### 2.4.5. Job Performance

Three items from the multilevel performance scale proposed by Griffin, Neal and Parker [78] were used to measure task performance proficiency, team member task proficiency and organization member. Participants were asked to indicate how often they had carried out the described behavior over the past month on a 5-point scale ranging from very little to a great deal. Items were: “Carried out the core parts of your job well”; “Coordinated your work with coworkers”; “Presented a positive image of the organization to other people”. Cronbach’s alpha was 0.77.

#### 2.4.6. Control Variables

Four control variables were considered based on theoretical and empirical evidence of their relationship to the variables included in our hypothesized model. Specifically, single-item measures of the number of hours worked per week, gender, age, and organizational tenure were all used as control variables in initial analyses. However, the inclusion of these control variables did not substantively change any of the results; thus, only results not including control variables are presented here. Analyses including these controls are available upon request.

## 3. Results

Means, standard deviations, reliabilities, and intercorrelations among study variables are displayed in Table 1. Hypotheses were tested with regression-based structural equation modeling in Mplus Version 8.3 (Muthén & Muthén, Los Angeles, CA, USA) [79] using full-information maximum likelihood estimation. Procedures for testing conditional and moderated moderated mediation outlined by Hayes [80] were followed using 10,000 bootstrapped samples to calculate 95% confidence intervals. All variables were mean-centered for the analyses [81,82]. All significant interaction effects were probed at scale values associated with the 20th, 50th, and 80th percentile [83]. Hypothesis 1 predicted that workaholism would mediate the relationship between workload and job performance, such that workload positively relates to workaholism, which, in turn, negatively relates to job performance. As shown in Table 2, workload had a nonsignificant relationship with workaholism (B = −0.004, *p* = 0.952) and workaholism had a nonsignificant negative relationship with job performance (B = −0.110, *p* = 0.059). Additionally, holding perfectionistic concerns and work engagement constant at their mean levels, the indirect effect of workload on job performance through workaholism was nonsignificant (estimate = 0.000, 95% bootstrap CI: −0.014 to 0.024). Thus, Hypothesis 1 was not supported. 

**Figure 1:**
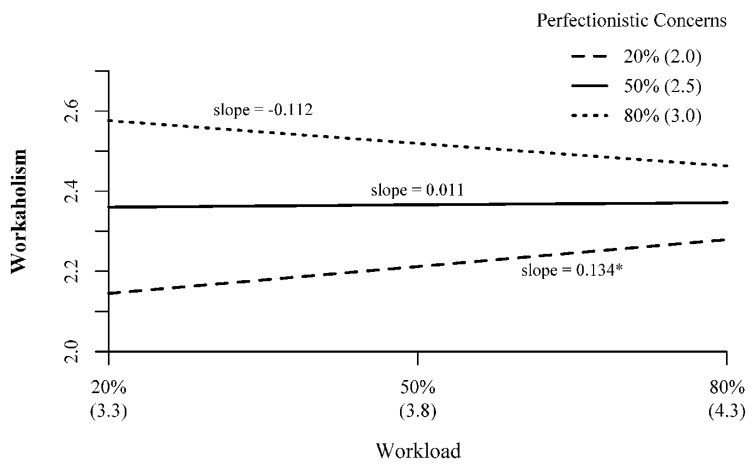
Interaction Effects of Workload and Perfectionistic Concerns on Workaholics; Note 20% = 20th percentile; 50% = 50th percentile; 80% = 80th percentile. Numbers in parentheses represent scale scores corresponding to the percentile. All measures on scales from 1–5. *—*p* < 0.05.

Hypothesis 2 proposed that the relationship between workload and workaholism is moderated by perfectionistic concerns such that the positive relationship would be stronger at higher levels of perfectionist concerns. Perfectionistic concerns significantly moderated the relationship between workload and workaholism (B = −0.247, *p* = 0.005). However, probing the interaction at the 20th, 50th, and 80th percentiles of perfectionistic concerns revealed that at higher levels of perfectionistic concerns (i.e., 80th percentile, scale score = 3.0/5) there was a negative—though nonsignificant—relationship between workload and workaholism (see Figure 1). Conversely, at lower levels of perfectionistic concerns (i.e., 20th percentile, scale score = 2.0/5) there was a significantly positive relationship between workload and workaholism (simple slope = 0.134, *p* = 0.036). In other words, when participants had lower levels of perfectionistic concerns, levels of workaholism increased as perceptions of workload increased. Taken together, while perfectionistic concerns did moderate the workload–workaholism relationship, it did so in the opposite direction as expected, not supporting Hypothesis 2.

**Figure 2:**
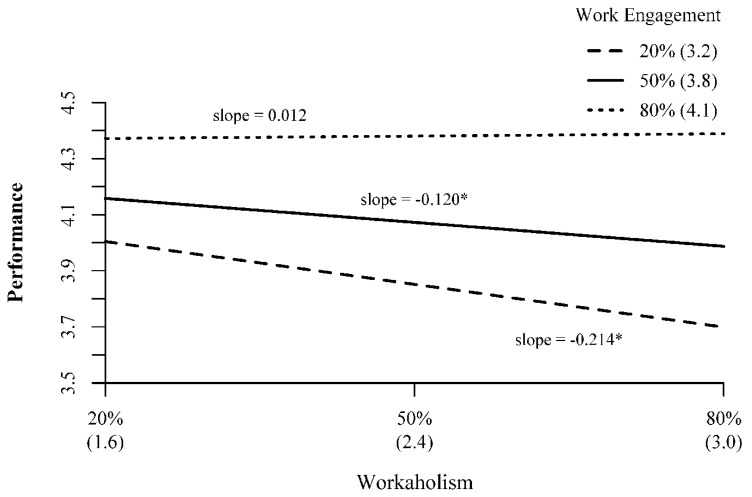
Interaction Effects of Workaholism and Work Engagement on Performance; Note 20% = 20th percentile; 50% = 50th percentile; 80% = 80th percentile. Numbers in parentheses represent scale scores corresponding to the percentile. All measures on scales from 1–5. *—*p* < 0.05.

Hypothesis 3 stated that the negative relationship between workaholism and job performance would be buffered by work engagement, such that higher levels of engagement would weaken the negative relationship between workaholism and job performance. In support of this hypothesis, work engagement significantly moderated the workaholism–performance relationship (B = 0.168, *p* = 0.042). Examining Figure 2, we see that at lower (i.e., 20th percentile, scale score = 3.2/5) and average (i.e., 50th percentile, scale score = 3.8/5) levels of work engagement, there is a significant and negative relationship between workaholism and job performance (20% simple slope = −0.214, *p* = 0.024; 50% simple slope = −0.120, *p* = 0.049). However, at higher levels of work engagement (i.e., 80th percentile, scale score = 4.1/5), there is a nonsignificant relationship between workaholism and job performance (80% simple slope = 0.012, *p* = 0.842). As such, Hypothesis 3 was fully supported.

For the indirect effect tested in Hypothesis 1, because all variables were mean-centered, the value for both moderators was the respective means. However, it is possible that these moderators affect the indirect effect independently or jointly. Because we hypothesize and examine an indirect effect in a model that has a moderator for each path of the indirect effect, we cannot ignore these moderators in relation to the indirect effect. Indeed, because the indirect effect is a function of two moderators, we cannot calculate or form an inference about the indirect effect without choosing values for each moderator [80]. In a first and second stage dual-moderated mediation model such as this, Hayes [80] advises before making inferences about independent moderated mediation of each moderator, one needs to test if the two moderators interact in their influence on the indirect effect. In other words, we need to test if the moderation of the indirect effect by one moderator is itself conditioned on the other moderator. Hayes calculates this effect with what he labels the index of moderated moderated mediation. With affirmative evidence of this interactive effect, the moderation of the moderated mediation can be probed to piece together a substantive interpretation of the results. Unfortunately, theory and research are unavailable at this level of nuance between the variables in this study. Thus, we put forth the following research questions:

R1: Do perfectionistic concerns and work engagement interact with each other in the moderation of the indirect effect (i.e., moderated moderated mediation)?

R2: If there is evidence of moderated moderated mediation (R1), is the moderated mediation conditional on (a) perfectionistic concerns or (b) work engagement (i.e., conditional moderated mediation)?

The first research question asks whether there is evidence of moderated moderated mediation. As shown in Table 2, our model provides support for moderated moderated mediation, with the index value of −0.042 and the 95% bootstrap confidence interval completely below zero (−0.111 to −0.006). Thus, in support of Research Question 1, perfectionistic concerns and work engagement interact with each other in the moderation of the indirect effect. With evidence of moderated moderated mediation, we can turn to Research Question 2, which asks whether the moderated indirect effect is conditioned on a) perfectionistic concerns or b) work engagement.

**Figure 3:**
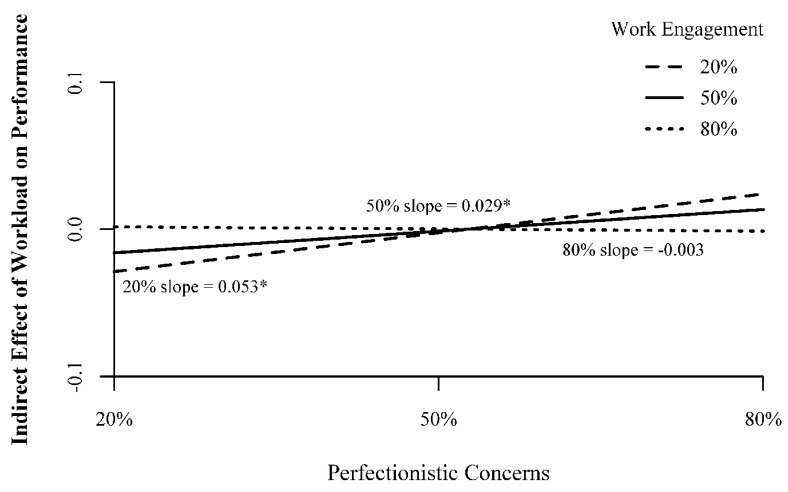
Conditional Moderated Mediation by Perfectionistic Concerns; Note 20% = 20th percentile; 50% = 50th percentile; 80% = 80th percentile. Numbers in parentheses represent scale scores corresponding to the percentile. All measures on scales from 1–5. *—95% bootstrap confidence interval does not include zero.

**Figure 4:**
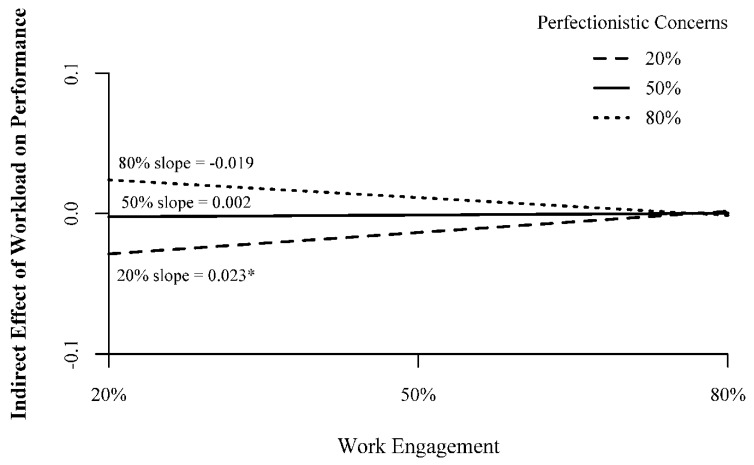
Conditional Moderated Mediation by Work Engagement. Note 20% = 20th percentile; 50% = 50th percentile; 80% = 80th percentile. Numbers in parentheses represent scale scores corresponding to the percentile. All measures on scales from 1–5. *—95% bootstrap confidence interval does not include zero.

Examining Table 2 and Figure 3 and Figure 4, we can see that perfectionistic concerns only moderates the indirect effect of workload on performance when work engagement is at lower or average levels (20% simple slope = 0.053, 95% bootstrap CI: 0.008–0.135; 50% simple slope = 0.029, 95% bootstrap CI: 0.002–0.081). At lower and average levels of work engagement, as perfectionistic concerns increase from lower to average to higher levels, the indirect effect of workload on job performance through workaholism goes from negative to nil to positive (Figure 3). In other words, it appears when employees are not as engaged with their work, higher levels of perfectionistic concerns improve the relationship between workload and performance, even to the point of creating a positive indirect effect of workload on performance.

On the other hand, work engagement only moderates the indirect effect from workload to performance when perfectionistic concerns levels are lower (20% simple slope = 0.023, 95% bootstrap CI: 0.002–0.070). With lower levels of perfectionistic concerns, as work engagement increases from lower to higher levels, the workload–job performance indirect effect changes from negative to nil. In other words, work engagement is only able to buffer the negative indirect effect of workload on performance when the employee does not show many perfectionistic concerns. Taken together, the answer to Research Question 2 is that both perfectionistic concerns and work engagement show evidence of significant conditional moderated mediation. It appears perfectionistic concerns and work engagement counteract the other’s influence on the relationship between workload, workaholism, and job performance. At higher levels of either or both moderators, the independent moderation effects of the indirect effect wash out. Thus, the counteracting forces of these moderators create nil relationships at the surface level but reveal a much more nuanced picture at deeper levels of inference.

## 4. Discussion

The current study tested a comprehensive first and second stage dual-moderated mediation model where workaholism mediated the interaction between workload and perfectionistic concerns on job performance, with the relationship between workaholism and job performance moderated by work engagement. We first examined the simple mediating effect of workaholism in the relationship between workload and job performance. These results were not supported. The non-significant relationship between workaholism and job performance, although it did not support our expectations, is in line with previous evidence also found by the meta-analysis of Clark and colleagues [11] and the latest contribution by Balducci and colleagues [21]. Regarding the relationship between workload and workaholism, contrary to our expectations and to some previous contributions [13,28], the results did not support the view implying a possible influence of workload on workaholism. However, Huyghebaert and colleagues [28] reported that workload was just related to the working excessively components of workaholism, whereas they did not find a significant influence of workload in predicting the working compulsively component of workaholism. As the current study used the behavioral addiction approach to the measure of workaholism, we offer a new piece of the puzzle for the understanding of the specific relationship between workload and workaholism.

Despite the incongruent evidence at the simple mediation level, the results offered much more interesting information when the moderators were taken into account. In particular, we tested the moderating effect of perfectionistic concerns in the relationship between workload and workaholism and the moderating effect of work engagement in the relationship between workaholism and job performance. Both the moderators were significant, although the moderating effect of perfectionistic concerns in the relationship between workload and workaholism was revealed to be significant in the opposite direction of our expectations. We expected that the relationship between workload and workaholism would have been stronger for individuals higher in perfectionistic concerns, whereas our results pointed out that the positive relationship between workload and workaholism was significant only at lower level of perfectionistic concerns. This may mean that only when a strong dispositional determinant of workaholism (i.e., perfectionism) is low, the role of environmental conditions become significant as potential antecedents of workaholism. However, we should note here that we only focused on perfectionistic concerns, defined as excessive concerns about making mistakes and chronic disappointment in the self for not living up to high-performance expectations.

To our knowledge, the interplay between workload and perfectionistic concerns was not tested before in relationship to workaholism. It is possible that individuals characterized by low levels of perfectionistic concerns, since they are not excessively concerned about making mistakes and they do not suffer from chronic disappointment in the self for not living up to high-performance expectations, could react to higher levels of workload by accepting it as challenge demands, activating a motivational process that would lead him/her to work even more at the level requested. Thus, it could be that the first gear of this mechanism might be adaptive and challenging, instead of a negative stressful condition, and only after they become workaholics, they would experience its detrimental outcomes. However, before elaborating any interpretation of this result, it should be noted that in the comprehensive model that we tested, we did not exclusively and separately test every single moderating effect, but all the variables were put together in the analysis, and thus every single relationship reflects also the adjustment of the whole model. As discussed later, the impact of each of the moderators is not assessed just on the simple relationship but also in the lens of its impact on the indirect effect and in the whole moderated moderated mediation model. Thus, we will supply further possible interpretations after discussing the moderating impact of work engagement and considering the two moderators together.

As far as the moderating role of work engagement, our hypothesis was fully supported; the moderation was in the direction we expected and in line with previous studies [14,69,70]. Specifically, at higher levels of work engagement, the relationship between workaholism and job performance was nonsignificant, whereas, at the medium and lower levels of work engagement, the negative relationship between workaholism and job performance was significant. Thus, when work engagement is lower, a stronger negative relationship between workaholism and job performance exists. In other words, it would seem that the lower level of work engagement would represent an essential condition for the negative relationship between workaholism and job performance to be significant. According to previous person-centered approaches to the study of the interplay between work engagement and workaholism by Gillet and colleagues [71], we could assume that disengaged workaholics could be at higher risk for underperforming at work. Moreover, the presence of work engagement as a moderator in the relationship between workaholism and job performance would explain the results obtained in previous studies revealing a nil relationship [11,21]. Taken together, although in the current study simple mediation analysis (i.e., perfectionistic concerns and work engagement held at their respective means) did not support our expectations, when the moderators were examined at a different level in the analysis, the results showed a more nuanced set of relationships among the variables.

As mentioned in the results section, the impact of the two moderators was assessed using the moderated moderated mediation approach [80], which strengthens our findings. The results of our research questions demonstrated that the index of moderated moderated mediation was significant and, thus, perfectionistic concerns and work engagement interact with each other in their influence of the indirect effect of workload on job performance via workaholism. Specifically, the two moderators counteract each other’s influence on the relationship between workload, workaholism, and job performance. In fact, all the paths, including the indirect path, of the tested model were significant at lower levels of the two moderators. This result speaks directly to the inconsistent findings in the literature surrounding these variables. This study demonstrates that without explicitly measuring and modeling both perfectionistic concerns and work engagement, the relationships among workload, workaholism, and job performance can be negative, positive, or nil. Thus, individual studies may find contradictory results from one another due to their specific sample levels of perfectionistic concerns and work engagement. Further, when combined into a meta-analysis, the mix of directions results in nil relationships.

### 4.1. Limitations and Future Directions

Although the current study reported original and interesting results, there are several limitations to be taken into account. First, regarding the conceptualization of workaholism, we should emphasize that we focused on a work addiction approach using the BWAS, which assesses each of the seven elements of work addiction with a single item. While this approach is useful for detecting the overall phenomenon in the workplaces and provides some clues for implementing a tailored clinical intervention [84], it limits our ability to examine dimensions of workaholism separately. Given the fact that workaholism is a very complex phenomenon, characterized by motivational, cognitive, emotional, and behavioral components [20,49,50,84,85,86], the measure used here might not be suitable for taking into account all the different nuances of workaholism. In fact, several empirical studies have found that perfectionism was more strongly associated with the cognitive dimension of workaholism (i.e., being driven to work), [22,87,88], whereas workload was more strongly associated with its behavioral dimension (i.e., working excessively), [28,50,85]. Thus, since a multifactorial scale for assessing workaholism (including motivational, cognitive, emotional, and behavioral dimensions) has been recently developed by Clark and colleagues [84], it would be interesting in the future to assess the model in the current study considering the four dimensions of this new scale. Doing so would determine which specific aspects of workaholism may be the driving factors of the relationships studied here. Finally, we examined overall work engagement in our model rather than the separate dimensions of vigor, dedication, and absorption. We took this approach to limit model complexity and based on recommendations for the measurement of work engagement [77,89]. However, recent research [90] indicates dimensions of work engagement may have differential relationships with workaholism; thus, future research in this area should also examine the role of specific engagement dimensions.

Second, we tested a two-wave model where all the variables were measured at T1, except for job performance that was measured at T2 one month later. While the separation of independent and dependent variables helps establish temporal precedence and reduces common method variance, other research designs should be used to better establish causality. Moreover, the measure of job performance after one month could be useful for testing the impact of the other variables considered in the study on this specific variable, this method is still problematic for testing the causal inference among the variables measured at T1 and, thus, results should be interpreted with caution. For instance, we found that the moderating role of perfectionistic concerns in the relationship between workload and workaholism was significant, but in the opposite direction of our expectations. Although theoretically founded, we should interpret this specific result with caution. In particular, to clearly address the issue of the reciprocal influence between workload and workaholism, a cross-lagged model could be recommended in a future study including both the two variables at two different times of data collection. Although Huyghebaert and colleagues [28] tested a cross-lagged model including workload and workaholism, they only tested the impact of workload on workaholism, neglecting the possible impact of workaholism on workload. Since literature would suggest a reciprocal influence, it would be important to test a cross-lagged model considering both the reciprocal impact of the two variables in a certain period of time. More properly, a three-waves study should be recommended for adequately capturing the mediation model, measuring the antecedents at T1, the mediator at T2, and the outcomes at T3.

Third, we use self-reported questionnaires for the data collection, and this creates the risk of common method variance. According to Podsakoff, MacKenzie, Lee and Podsakoff [91], common method variance problems could be reduced in different ways, such as by guaranteeing the anonymity of the survey and by instructing participants that there are no right or wrong answers in the questionnaire items. We followed both of these suggestions. However, in future studies, an effort should be made for collecting objective data, especially for job performance. This limitation of the self-report measures might be particularly inadequate for behavioral measures, such as performance, since, in general, individuals appear to be biased toward judging their own behavior as meeting higher standards than the behavior of others [92].

Finally, the moderated moderated mediation model hypothesized and tested in this study is quite complex. While a sample size over 200 is typical in workaholism and work addiction research, larger sample sizes may be required to ensure adequate statistical power by contemporary standards. Additionally, it is essential that the effects found here are replicated before strong conclusions are drawn regarding the complex relationship between workload, workaholism, perfectionism, work engagement, and job performance. Thus, while the present study highlights a nuanced relationship among these variables, as with all complex models, we recommend future research on this topic to cross-validate these findings and that the conclusions of the present study be interpreted with caution pending replication.

### 4.2. Practical Implications

Results from the current study offer several useful implications for managers and practitioners with regard to the link between workload and workaholism and job performance. Although our results did not reveal a significant connection between these variables at the surface, we found that a more nuanced process might exist, including perfectionistic concerns and work engagement. In particular, work engagement seems to be a crucial variable to be taken into consideration in this process. Thus, managers and practitioners should monitor the work engagement levels in situations where workload is necessary and unavoidable. Ideally, overworking should always be avoided. However, it is likely that in certain circumstances (e.g., during times of organizational changes or downsizing, in a very competitive organizational climate), workload can be particularly high. In these cases, managers should be aware that work engagement could buffer the possible detrimental effect of the chain connecting workload and workaholism to lower levels of job performance. Work engagement can be engendered by promoting healthy and happy workplace climates, where individuals can develop their careers in a sustainable way [93]. In this regard, work–life balance interventions could be an option to address the needs of workers by reducing the working time and gaining time to spend in nonwork activities, increasing both work engagement and job performance [94]. In particular, leaders, who serve as a liaison between employees and management, should be trained to manage the situations where both workload and the risk of increasing the level of workaholism are particularly high.

Regarding the role of perfectionistic concerns, our findings showed that lower levels of this individual difference variable might have a role in the tested model. Thus, managers should be aware that the first gear of this mechanism could be perceived as a challenge for these individuals, but it is possible that in a second stage, they would develop workaholism. Future studies should better clarify this issue, in order to prevent work addiction and promote healthy work contexts [9]. It is detrimental in modern society that working for many hours is considered socially acceptable and often results in recognition and gratification. As such, it can be difficult to detect and address workaholism. Managers should be aware of the differences between workaholism and work engagement and be alerted of the risk of inducing workaholism, instead of work engagement, by increasing, for instance, workload. More generally, it is the authors’ view that a deep change in society should be undertaken for promoting healthy and efficient workplaces, taking into account sustainable work–life balance interventions. It is our hope that this study moves us in that direction by clarifying the complex relationship between workload, workaholism, and job performance, providing target-points for effective workplace interventions.

## 5. Conclusions

The current study showed that the relationship among workload, workaholism, and job performance could be better explained through the complex interplay of perfectionistic concerns and work engagement in this mediated relationship.

## Figures and Tables

**Table 1:** Descriptives, inter-correlations and reliabilities of the study variables.

Variable	Mean	SD	1	2	3	4	5	6	7	8	9
1. Age	43.9	11.5									
2. Gender			−0.05								
3. Working hours x week	42.46	10.7	−0.06	−0.17 *							
4. Tenure	15.5	11.2	−0.20 **	0.11	0.01						
5. Workaholism T1	2.34	0.71	0.01	−0.10	0.24 **	0.01	0.78				
6. Perfectionistic concerns T1	2.56	0.74	0.14 *	−0.09	0.09	0.08	0.32 **	0.84			
7. Work engagement T1	3.83	0.69	0.01	0.01	0.13	0.07	−0.16 *	−0.15 *	0.90		
8. Workload T1	3.77	0.75	−0.03	0.07	0.14 *	0.02	0.15 *	0.26 **	0.11	0.78	
9. Performance T2	4.07	0.66	−0.13	0.01	0.03	0.10	−0.15 *	−0.10	0.45 **	0.23 **	0.77

Note: **—*p* < 0.01;*—*p* < 0.05.

**Table 2:** Results of first and second stage dual-moderated mediation model.

Parameter	Coefficient	Outcome		
		Workaholism (*SE*)	Coefficient	Performance (*SE*)
Intercept		2.385 (0.046)		4.087 (0.040)
Main effects				
Workload	*a*_1_	−0.004 (0.072)		
Workaholism			*b*_1_	−0.110 (0.058)
Perfectionistic concerns	*a*_2_	0.322 *** (0.071)		
Work engagement			*b*_2_	0.405 *** (0.059)
Interaction effects				
Workload × perfectionistic concerns	*a*_3_	−0.247 ** (0.088)		
Workaholism × work engagement			*b*_3_	0.168 * (0.083)
	*R* ^2^	0.168	*R* ^2^	0.212
				
			Index	95% bootstrap CI
Indirect effect ^a^			0.000	−0.014 to 0.024
Moderated moderated mediation			−0.042	−0.111 to −0.006
Conditional moderated mediation				
By perfectionistic concerns at	20% Engagement (3.2)		0.053	0.008 to 0.135
	50% Engagement (3.8)		0.029	0.002 to 0.081
	80% Engagement (4.6)		−0.003	−0.034 to 0.025
By work engagement at	20% Perfectionistic concerns (2.0)		0.023	0.002 to 0.070
	50% Perfectionistic concerns (2.5)		0.002	−0.024 to 0.031
	80% Perfectionistic concerns (3.0)		−0.019	−0.078 to 0.008

Note: SE — standard error. CI — confidence interval. ^a^—Holding perfectionistic concerns and work engagement constant at their mean values. All estimates are unstandardized from analyses with all variables centered; for ease of interpretation, intercepts and scale scores are in original scale units rather than centered units. 20% = 20th percentile; 50% = 50th percentile; 80% = 80th percentile. Numbers in parentheses next to % represent scale scores corresponding to the percentile. All measures on scales from 1–5; *—*p* < 0.05 **—*p* < 0.01 ***—*p* < 0.001.
